# Origin and evolution of *BZR* gene family in plants, pan-genome analysis of the *BZR1* gene family, and functional characterization of *CaBZR1.2* in pepper lateral branch development

**DOI:** 10.1093/hr/uhag015

**Published:** 2026-01-20

**Authors:** Wujun Xing, Dan Zhang, Peiru Li, Qingzhi Cui, Xinyi Huang, Lianzhen Mao, Yanan Zhao, Jingwei Duan, Yanlong Li, Sha Yang, Cheng Xiong, Xuexiao Zou, Xiongze Dai, Lijun Ou, Zhoubin Liu

**Affiliations:** Key Laboratory for Vegetable Biology of Hunan Province, Engineering Research Center for Horticultural Crop Germplasm Creation and New Variety Breeding, Ministry of Education, College of Horticulture, Hunan Agricultural University, Changsha 410125, China; Vegetable Variety Creation Center, Yuelushan Laboratory, Changsha 410128, China; Key Laboratory for Vegetable Biology of Hunan Province, Engineering Research Center for Horticultural Crop Germplasm Creation and New Variety Breeding, Ministry of Education, College of Horticulture, Hunan Agricultural University, Changsha 410125, China; Vegetable Variety Creation Center, Yuelushan Laboratory, Changsha 410128, China; Key Laboratory for Vegetable Biology of Hunan Province, Engineering Research Center for Horticultural Crop Germplasm Creation and New Variety Breeding, Ministry of Education, College of Horticulture, Hunan Agricultural University, Changsha 410125, China; Vegetable Variety Creation Center, Yuelushan Laboratory, Changsha 410128, China; Key Laboratory for Vegetable Biology of Hunan Province, Engineering Research Center for Horticultural Crop Germplasm Creation and New Variety Breeding, Ministry of Education, College of Horticulture, Hunan Agricultural University, Changsha 410125, China; Vegetable Variety Creation Center, Yuelushan Laboratory, Changsha 410128, China; Key Laboratory for Vegetable Biology of Hunan Province, Engineering Research Center for Horticultural Crop Germplasm Creation and New Variety Breeding, Ministry of Education, College of Horticulture, Hunan Agricultural University, Changsha 410125, China; Vegetable Variety Creation Center, Yuelushan Laboratory, Changsha 410128, China; Key Laboratory for Vegetable Biology of Hunan Province, Engineering Research Center for Horticultural Crop Germplasm Creation and New Variety Breeding, Ministry of Education, College of Horticulture, Hunan Agricultural University, Changsha 410125, China; College of Landscape Architecture and Horticulture Science, Southwest Forestry University, Kunming 650000, China; Key Laboratory for Vegetable Biology of Hunan Province, Engineering Research Center for Horticultural Crop Germplasm Creation and New Variety Breeding, Ministry of Education, College of Horticulture, Hunan Agricultural University, Changsha 410125, China; Vegetable Variety Creation Center, Yuelushan Laboratory, Changsha 410128, China; Key Laboratory for Vegetable Biology of Hunan Province, Engineering Research Center for Horticultural Crop Germplasm Creation and New Variety Breeding, Ministry of Education, College of Horticulture, Hunan Agricultural University, Changsha 410125, China; Vegetable Variety Creation Center, Yuelushan Laboratory, Changsha 410128, China; Key Laboratory for Vegetable Biology of Hunan Province, Engineering Research Center for Horticultural Crop Germplasm Creation and New Variety Breeding, Ministry of Education, College of Horticulture, Hunan Agricultural University, Changsha 410125, China; Vegetable Variety Creation Center, Yuelushan Laboratory, Changsha 410128, China; Key Laboratory for Vegetable Biology of Hunan Province, Engineering Research Center for Horticultural Crop Germplasm Creation and New Variety Breeding, Ministry of Education, College of Horticulture, Hunan Agricultural University, Changsha 410125, China; Vegetable Variety Creation Center, Yuelushan Laboratory, Changsha 410128, China; Key Laboratory for Vegetable Biology of Hunan Province, Engineering Research Center for Horticultural Crop Germplasm Creation and New Variety Breeding, Ministry of Education, College of Horticulture, Hunan Agricultural University, Changsha 410125, China; Vegetable Variety Creation Center, Yuelushan Laboratory, Changsha 410128, China; Key Laboratory for Vegetable Biology of Hunan Province, Engineering Research Center for Horticultural Crop Germplasm Creation and New Variety Breeding, Ministry of Education, College of Horticulture, Hunan Agricultural University, Changsha 410125, China; Vegetable Variety Creation Center, Yuelushan Laboratory, Changsha 410128, China; Key Laboratory for Vegetable Biology of Hunan Province, Engineering Research Center for Horticultural Crop Germplasm Creation and New Variety Breeding, Ministry of Education, College of Horticulture, Hunan Agricultural University, Changsha 410125, China; Vegetable Variety Creation Center, Yuelushan Laboratory, Changsha 410128, China; Key Laboratory for Vegetable Biology of Hunan Province, Engineering Research Center for Horticultural Crop Germplasm Creation and New Variety Breeding, Ministry of Education, College of Horticulture, Hunan Agricultural University, Changsha 410125, China; Vegetable Variety Creation Center, Yuelushan Laboratory, Changsha 410128, China; Key Laboratory for Vegetable Biology of Hunan Province, Engineering Research Center for Horticultural Crop Germplasm Creation and New Variety Breeding, Ministry of Education, College of Horticulture, Hunan Agricultural University, Changsha 410125, China; Vegetable Variety Creation Center, Yuelushan Laboratory, Changsha 410128, China

## Abstract

The transcription factor *BRASSINAZOLE-RESISTANT 1* (*BZR1*) plays a crucial role not only in plant responses to various biotic and abiotic stresses but also serves a critical function in plant growth and development. In this study, we analyzed the origin and evolution of the *BZR* family in plants. Then, we identified nine *CaBZR1* genes from the pepper pan-genome and performed bioinformatics analyses. Through the integration of transcriptome data analysis with our prior bioinformatics findings, we have identified and selected a specific member of the *CaBZR1* family, *CaBZR1.2*, for further comprehensive investigation. We systematically investigated the biological function of *CaBZR1.2* in pepper through classical reverse genetics approaches and subsequently identified proteins that interact with CaBZR1.2. After inhibiting the expression of *CaBZR1.2* via virus-induced gene silencing (VIGS), the growth of pepper lateral branches was significantly suppressed, whereas heterologous overexpression of *CaBZR1.2* increased lateral branch number in tomato. This result confirms the key role of *CaBZR1.2* in the development of pepper lateral branches. Furthermore, protein–protein interaction assays confirmed that the Sucrose Nonfermenting 1-Related Protein Kinase 1 β subunit 2 (CaSnRK1β2) protein interacts with CaBZR1.2, with subsequent analyses revealing that these two proteins modulate pepper lateral branch development through a mutually antagonistic regulatory mechanism. This study reveals a novel mechanism by which *CaBZR1.2* and *CaSnRK1β2* coordinately regulate lateral branch development in pepper, providing candidate genes and a theoretical basis for the molecular breeding for pepper plant architecture.

## Introduction

Lateral branches constitute a critical component of plant architecture, significantly influencing crop yield and production costs [1]. In cereal crops like rice and wheat, tiller number is a decisive factor for final yield, which is similarly applicable to horticultural crops [2, 3]. However, in the cultivation of horticultural crops such as peppers, tomatoes, and cucumbers, lateral branches frequently require timely and appropriate manual pruning, thereby escalating production costs. Consequently, understanding the regulatory mechanisms governing lateral branch development and optimizing branch architecture are of great agronomic significance for breeding crop varieties with optimal branch architectures.

Lateral branches originate from specific undifferentiated meristematic tissues in the leaf axils, known as axillary meristems (AMs). These AMs first develop into axillary buds, which then form lateral branches [4]. Therefore, the development process of lateral branches can be divided into two stages: AM initiation and axillary bud growth [5]. Lateral branching is a highly plastic process. Under favorable growth conditions, some axillary buds grow into lateral branches after breaking dormancy; under adverse conditions, they remain dormant [6]. This process is controlled by multiple hormonal signaling pathways, including auxin, cytokinins (CKs), and strigolactones (SLs) [7]. However, few studies have explored how brassinosteroids (BRs) regulate branching, and the interactions between BRs, other core hormonal signaling pathways, and sugar signaling pathways in the branching regulatory network remain poorly understood. BRs are a class of polyhydroxylated steroidal plant hormones that play a pivotal, evolutionarily conserved role in regulating cellular processes and plant development [8]. Through their dedicated signal transduction cascades, BRs precisely modulate the activity of two core transcription factors—*BZR1* and *BES1*—and thereby regulate thousands of target genes involved in various developmental processes [9, 10]. Previous studies have shown that *BES1/BZR1* is involved in numerous biological processes, such as shoot apical development [11], stomatal development [12], seed development [13], seed germination [14, 15], reproductive development [16], plant morphogenesis [14, 17–19], chlorophyll metabolism [20], fruit morphogenesis [21], and fruit ripening [22]. Additionally, *BES1/BZR1* genes can be induced by drought, cold, heat, salt stress [23–26], and nitrogen starvation [27]. To date, the mechanism by which BRs regulate branching remains poorly understood. Recent studies have demonstrated that *BES1/BZR1* is an important regulator in the branching/tillering process [28, 29]. For example, rice *OsBZR1* and *Arabidopsis BES1* differentially regulate shoot branching in these two species based on their specific integration of BR signals [29]. In BR signaling, *BZR1* directly targets and represses the expression of *CUC3*, thereby affecting the expression of *LAS* and inhibiting axillary bud formation [30]. A recent study reported the function of *BZR1* in alfalfa, which has the function of promoting branching in *Medicago truncatula* and *Medicago sativa* [31].

Pepper is a vegetable crop cultivated worldwide with great nutritional and economic value [32]. The number of lateral branches varies greatly among different types of peppers. The demand for the number of lateral branches also differs during the production process in pepper cultivation. For example, for some fresh eating-type large-sized peppers, a smaller number of lateral branches is required to ensure a reasonable planting density and achieve high yield. While small fruit-type peppers especially the clustered pod peppers require more side branches to achieve high yields. But currently, there are relatively few studies on the regulatory mechanisms of lateral branch development in peppers, and the function of the *BZR1* family in pepper remains largely unknown. Therefore, conducting a comprehensive genomic and expression analysis of this family is extremely necessary for studying the regulatory mechanism of pepper branching.

Herein, we investigated the origin and evolutionary trajectory of the *BZR* gene in plants. Then, through genome-wide screening combined with bioinformatics analysis, we identified a transcription factor, *CaBZR1.2*, and demonstrated that it plays a positive regulatory role in axillary bud growth in pepper. Furthermore, we combined yeast two-hybrid (Y2H), bimolecular fluorescence complementation (BiFC), and luciferase complementation imaging (LCI) assays to systematically verify that CaBZR1.2 interacts with CaSnRK1β2 at the protein level. This interaction modulates axillary bud outgrowth to regulate lateral branch growth in pepper. In summary, these findings provide significant insights and offer new clues for elucidating the molecular mechanisms underlying branching development in pepper.

## Results

### Identification and distribution of *BZR* gene family members in various plant lineages

In this study, we identified 566 *BZR* gene family members in total from 78 species, including glaucophytes (1), prasinodermophytes (1), red algae (3), green algae (18), charophytes (6), bryophytes (3), pteridophytes (2), gymnosperms (5), monocots (5), and dicots (34). The specific number of *BZR* genes in each species and group is detailed in Tables S1 and S2.

Among algal plants, *BZR* family members were only identified in the Charophyta (Table S2), with *Spirogloea muscicola* containing five *BZR* genes, the highest number among all algal species. In bryophytes, the number of *BZR* genes is highly consistent across different species. For instance, *Marchantia polymorpha*, *Fontinalis antipyretica*, and *Entodon seductrix* each contain four *BZR* genes, indicating that the *BZR* family size is relatively conserved in this lineage. In pteridophytes, with the enhancement of plants’ adaptation to terrestrial environments, the number of *BZR* genes expanded significantly. *Alsophila spinulosa* was identified with 18 *BZR* genes, the highest among ferns, and even the fern species with the fewest copies contained nine *BZR* genes. In gymnosperms, the number of *BZR* family members ranges from five to 12. For instance, *Abies alba* contains 12, whereas *Gnetum montanum* contains only five. In angiosperms, the family size expanded further. The number of *BZR* genes in monocots ranges from six in *Oryza sativa* to 43 in *Triticum turgidum*. Among dicots, *Gossypium raimondii* has the most, with 42 *BZR* genes, while *Fragaria nilgerrensis* and *Beta vulgaris* contain only five (Table S2). Collectively, the *BZR* family exhibits significant lineage-specific expansion patterns in terrestrial plants, particularly in angiosperms.

### Phylogenetic and evolutionary trajectory analysis of the *BZR* gene family

**Figure 1:**
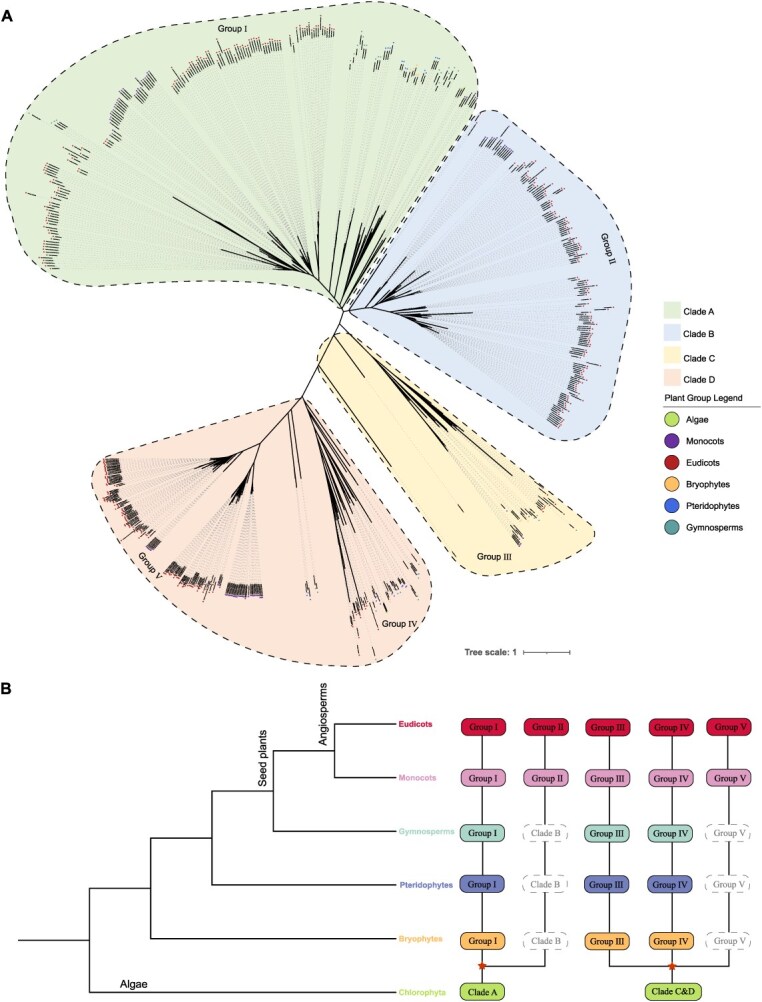
Phylogenetic analysis and ancient evolutionary trajectory of *BZR* gene family in plants. (A) Construction of a phylogenetic tree using the protein sequences of all the *BZR* family genes from 78 species. (B) Evolutionary trajectory of *BZR* genes in plants. The six major plant lineages are represented by different colors, solid round rectangles indicate the presence of *BZR* family members in the corresponding plant lineages, and dashed rectangles suggest the absence of genes due to gene losses. Inferred ancient gene duplications are depicted as pentacles.

To analyze the phylogenetic relationships of the *BZR* gene family across different plant lineages, we constructed a phylogenetic tree including all *BZR* members based on 78 species (Fig. 1A) and divided it into four evolutionary clades (clade A, B, C, and D) based on tree topology. The results revealed that *BZR* genes in Charophyta species are distributed in clades A, C, and D, indicating that the *BZR* family underwent multiple early divergences during the Charophyta stage. *BZR* genes in pteridophytes and gymnosperms are primarily clustered in clades A, C, and group IV (subgroup of clade D), while *BZR* genes in monocots and dicots are distributed across all subgroups. Notably, clade B and the group V subgroup of clade D only contain *BZR* genes from monocots and dicots, suggesting these subgroups are either angiosperm-specific or underwent significant expansion in angiosperms.

To further trace the evolutionary history of the *BZR* gene family in plant lineages, we combined the phylogenetic tree with species system relationships to create evolutionary diagrams for each group, thereby reconstructing the evolutionary trajectory of the *BZR* family (Fig. 1B). The results indicate that the *BZR* family originated in the Charophyta stage and differentiated into two ancient lineages, clade A and clade C & D. As plants transitioned from aquatic to terrestrial environments, clade A evolved into group I in bryophytes, while clade C & D expanded and formed the precursors of group III and group IV. In the pteridophyte and gymnosperm stages, group I/III/IV remained stable across lineages, while *BZR* gene duplication in group V of clade B and clade D showed leaps, indicating that the basic structural framework of the *BZR* family was largely established at this time.

During the angiosperm stage, with increased gene duplication events and functional differentiation, the original group V in clade B and clade D further evolved into the typical group II and group V, resulting in the *BZR* family forming five stable subgroups (group I–V) in angiosperms, exhibiting varying degrees of lineage-specific expansion in both monocots and dicots. Collectively, the *BZR* gene family underwent a continuous evolutionary process of ‘origin in Charophyta—initial expansion in terrestrial plants—framework stabilization in vascular/seed plants—refinement in angiosperms’.

### Identification and phylogenetic analysis of the *BZR1* pan-genome family in pepper

**Figure 2:**
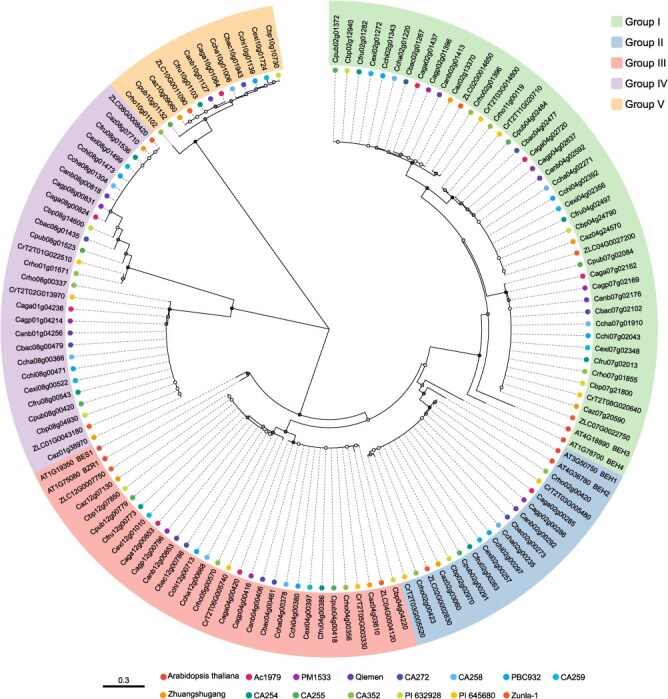
Phylogenetic analysis of *BZR1* family members between 14 *Capsicum* genomes and *A. thaliana*. The materials corresponding to these 14 *Capsicum* genomes cover diverse cultivated species and wild species of the *Capsicum* genus, with specific/variety species as follows: ‘Ac1979’ and ‘PM1533’, *C. annuum* var. Glabriusculum; ‘Qiemen’, ‘Zhuangshugang’ and ‘Zunla-1’, *C. annuum*; ‘CA272’ and ‘PI 632928’, *C. baccatum*; ‘CA258’, *C. chacoense*; ‘PBC932’, *C. chinense*; ‘CA259’, *C. eximium*; ‘CA254’, *C. frutescens*; ‘CA255’ and ‘PI 645680’, *C. pubescens*; ‘CA352’, *C. rhomboideum*.

Members of *CaBZR1* were identified from the pepper pan-genome using HMMER and BLASTp methods, and nine *CaBZR1* genes were ultimately identified, designated as *CaBZR1.1* to *CaBZR1.9*. To further investigate the functions and evolutionary characteristics of the pepper *BZR1* gene family, a phylogenetic tree was constructed using the BZR1 protein sequences of pepper and *Arabidopsis* (Fig. 2). Results revealed that the CaBZR1 proteins were divided into five groups: group I clustered with *Arabidopsis* BEH3 and BEH4 in one clade; group II clustered with BEH1 and BEH2 in one clade; group III clustered with BZR1 and BES1 in one clade; while group IV and V underwent independent evolution and formed distinct clades in the phylogenetic tree, suggesting potential functional divergence among these subgroups.

### Evolution of CaBZR1s

**Figure 3:**
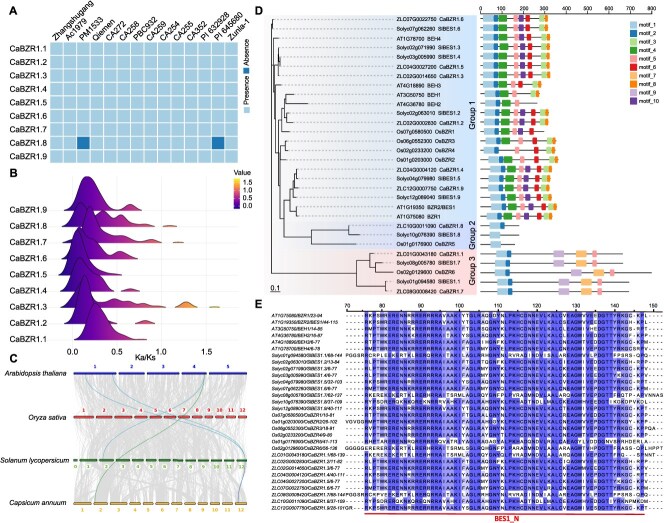
Evolution of CaBZR1s. (A) Heatmap illustrating the presence and absence of the nine *CaBZR1* genes in 14 peppers. (B) Distribution of Ka/Ks values of *CaBZR1s* in 14 pepper varieties. (C) Intergenomic synteny analysis between *A. thaliana* (*Arabidopsis*), *O. sativa* (rice), *Solanum lycopersicum* (tomato), and *C. annuum* (pepper). (D) Phylogenetic analysis and conserved motif analysis of BZR1 family genes in *Arabidopsis*, rice, tomato, and pepper. (E) Multiple sequence alignment of conserved domains of BZR1 family genes in *Arabidopsis*, rice, tomato, and pepper.

Presence–absence variation analysis revealed that *CaBZR1* is widely distributed among different peppers, with the *CaBZR1.8* gene absent only in two accessions, namely ‘PM1533’ and ‘PI645680’—a phenomenon that may be associated with genetic variation and deletion events (Fig. 3A). Ka/Ks analysis indicated that some *CaBZR1* genes in certain germplasms were under positive selection (Ka/Ks > 1), while all other genes are subject to purifying selection (Ka/Ks < 1), suggesting functional conservation (Fig. 3B).

To investigate the contraction and expansion of the *BZR1* gene family during evolution, we used Zunla-1 as the reference genome to analyze the collinearity of orthologous *BZR1* genes among pepper, *Arabidopsis*, rice, and tomato. The results showed that *CaBZR1.2* and *CaBZR1.9* are relatively conserved across different species (Fig. 3C).

Phylogenetic analysis was performed using BZR1 protein sequences from *Arabidopsis*, rice, tomato, and pepper revealed that all members of the CaBZR1 family contain the conserved BES1_N domain (Fig. 3D and E). Furthermore, MEME analysis was conducted to conserved motifs, and the *CaBZR1* members could be divided into three groups based on their motif compositions. Motif 1 was present in all members and was exactly located within the BES1_N domain. Members of group 1 exhibited a highly similar motif composition pattern, with most containing motifs 1, 2, 3, 4, 5, 6, 8, and 10. Among these, motifs 4 and 5 are rich in serine and serve as potential phosphorylation sites for GSK3 kinases; motif 8 contains an LXLXLX-type EAR domain, which possesses transcriptional repressive activity (Fig. 3D and Fig. S1). In contrast, members of group 2 and group 3 showed distinctly different motif compositions: group 2 members only contained the basic motif 1, with all other motifs absent. Group 3 harbored the specific motifs 7 and 9, which constitute a glycoside hydrolase family 14 domain. This suggests that group 3 members have undergone significant functional divergence and possess the potential to exert additional amylase-related functions.

### Expression pattern and subcellular localization of CaBZR1.2

**Figure 4:**
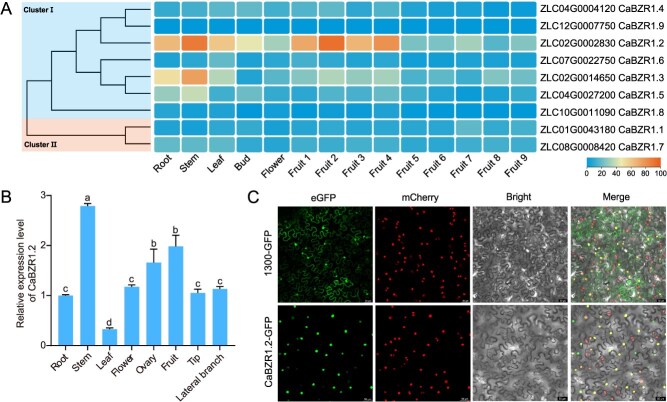
Expression analysis of *CaBZR1.2*. (A) Analysis of the expression of pepper *BZR1* family members in different tissues and developmental stages using RNA-seq data. Fruits 1–9 represent fruit lengths of 0–1, 1–3, 3–4, 4–5 cm, mature green stage, breaker stage, 3, 5, and 7 days postbreaker, respectively. (B) Analysis of the expression of *CaBZR1.2* in different tissues using RT-qPCR. (C) Subcellular localization of CaBZR1.2 in *N. benthamiana* leaves, scale bar, 50 μm.

Gene expression patterns are closely associated with biological function. First, based on published RNA-seq data [33], we generated a heatmap to visualize the expression patterns of *CaBZR1* family members in various tissues (Fig. 4A). Given that *CaBZR1.2* exhibited relatively high expression levels in diverse pepper tissues, we further validated the expression pattern of *CaBZR1.2* using quantitative real-time PCR (RT-qPCR) (Fig. 4B). The highest expression level was observed in stems; furthermore, *CaBZR1.2* also showed relatively high expression in ovaries and young fruits, which is consistent with the published transcriptome data. To investigate the subcellular localization of the CaBZR1.2 protein, we constructed a 35S:*CaBZR1.2*-GFP vector, and this fusion construct was transiently expressed in tobacco epidermal cells. The results showed that a strong fluorescent signal was detected in the nuclei of *Nicotiana benthamiana* leaves, indicating that CaBZR1.2 localizes to the nucleus in *N. benthamiana* leaves (Fig. 4C).

### Silencing of *CaBZR1.2* significantly inhibits lateral branch formation

**Figure 5:**
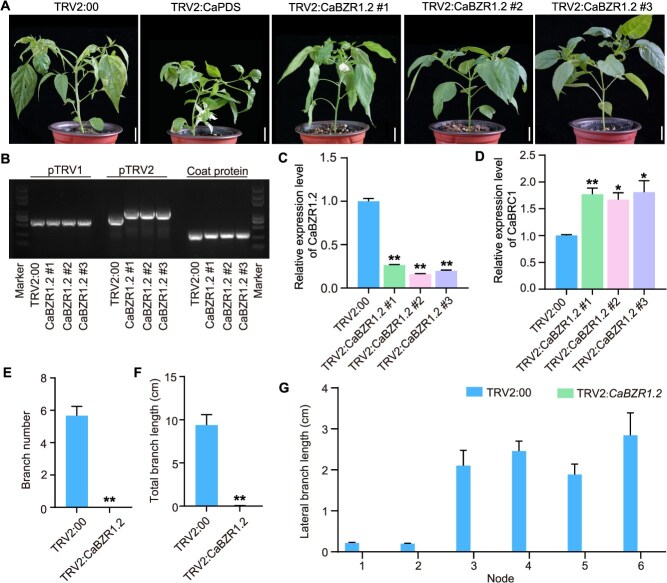
Gene silencing of *CaBZR1.2* inhibits lateral branch growth in pepper. (A) Representative images of *CaBZR1.2*-silenced lines, scale bars, 2 cm. (B) Identification of tobacco rattle virus (TRV) via PCR. The template was prepared by reverse transcription of total RNA extracted from the nodes and lateral branches of the negative control TRV2:00 and experimental groups TRV2:*CaBZR1.2*. (C and D) Expression level of *CaBZR1.2* and *CaBRC1* in *CaBZR1.2*-silenced plants. (E and F) Number of lateral branches and total lateral branch length in control and *CaBZR1.2*-silenced lines. (G) Branch length at each node of control and *CaBZR1.2*-silenced lines. Values are presented as mean ± SD (*n* = 3).

To further verify the relationship between *CaBZR1.2* expression and lateral branch development as well as the expression of related genes in pepper, we silenced the *CaBZR1.2* gene in ‘Zhangshugang’ pepper, and generated three independent *CaBZR1.2*-silenced lines (Fig. 5A and B). The results showed that the lateral branch length of *CaBZR1.2*-silenced lines was significantly shortened, and the number of lateral branches was significantly reduced (Fig. 5E and F). RT-qPCR was used to detect the expression level of *CaBZR1.2* in stems, the transcriptional level of *CaBZR1.2* in silenced lines decreased by 73.0%–85.0% compared with the control (Fig. 5C). *BRC1* is a key gene regulating lateral branch initiation [17, 28]. Here, we detected the transcriptional level of *CaBRC1*. Notably, its expression in silenced lines increased by 55.1%–103.3% compared with the control, which was opposite to the expression pattern of *CaBZR1.2* (Fig. 5D). These results indicate that *CaBZR1.2* plays a promoting role in the initiation and elongation of lateral branches in pepper.

### Overexpression of *CaBZR1.2* promotes lateral branch initiation and elongation

**Figure 6:**
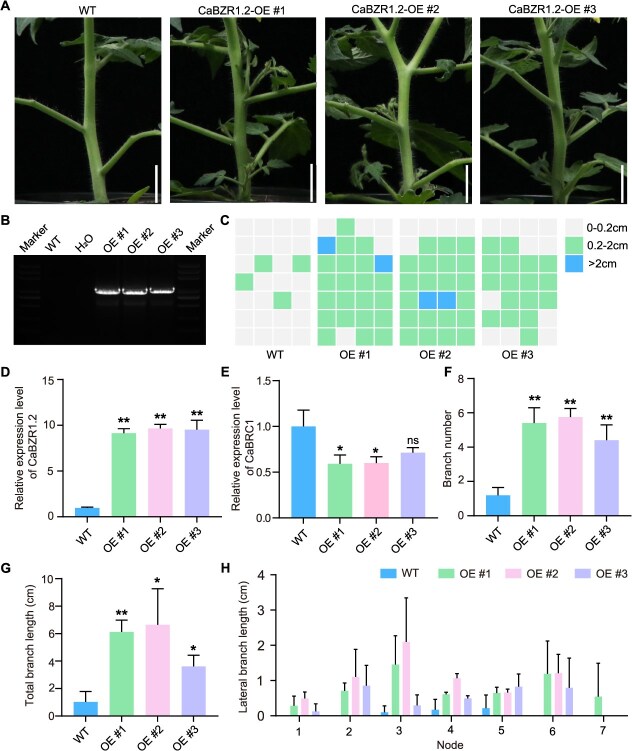
OE of *CaBZR1.2* promotes tomato lateral branch growth. (A) Representative images of heterologous OE of *CaBZR1.2* in tomato, Scale bars, 2 cm. (B) PCR identification of three OE lines. (C) Diagram data showing the growth position and length of lateral branches in wild type (WT) and *CaBZR1.2*-OE lines, each column represents an individual plant, each cell represents a node in the tomato plant. Gray squares indicate no bud growth, green squares indicate bud length between 0.2 and 2 cm, and blue squares indicate lateral branch length ≥2 cm. (D and E) Expression levels of *CaBZR1.2* and *CaBRC1* in WT and OE lines. (F and G) The charts respectively represent the number of lateral branches, the average branch length at each node, and the total length of lateral branches in WT and OE lines. Values are mean ± SD (*n* = 4).

To further explore the role of *CaBZR1.2* in pepper lateral branch growth, we constructed an overexpression (OE) vector for this gene. Given the current difficulty in genetic transformation of pepper, heterologous OE of *CaBZR1.2* was performed in tomato in this study. Through vector amplification and detection of expression levels, three independent positive OE lines were screened out (Fig. 6B and D). Observation of selected representative plants revealed that, compared with the control, the OE lines had a significantly increased number of lateral branches and significantly longer lateral branch length—phenotypes that were opposite to those of *CaBZR1.2*-silenced pepper plants (Fig. 6A, C, and  F–H). These results further indicate that *CaBZR1.2* plays a distinct role in regulating the initiation and elongation of lateral branches.

### Interaction between CaSnRK1β2 and CaBZR1.2

To further investigate the molecular mechanism of pepper lateral branch development mediated by *CaBZR1.2*, we performed Y2H screening of a pepper cDNA library to identify proteins that interact with CaBZR1.2. We obtained multiple clones, including those encoding known proteins such as BIN2 and 14-3-3 proteins. Among these, one clone encoding a protein belonging to the SNF1-related protein kinase family attracted our attention. Through BLAST search (against *Arabidopsis* gene *AT4G16360*), this gene was annotated as *CaSnRK1β2*. *CaSnRK1β2* encodes a protein consisting of 282 amino acids, with a calculated molecular weight of 31.3 kDa. Subcellular localization results show that CaSnRK1β2 is located in the nucleus and cell membrane (Fig. S2C). Based on our phylogenetic tree (Fig. S2A), homologs of the SnRK1 family from *Arabidopsis*, rice, and tomato were divided into three subgroups, CaSnRK1β2 is homologous to SnRK1β, which is directly or indirectly involved in BR signal transduction [34, 35].

**Figure 7:**
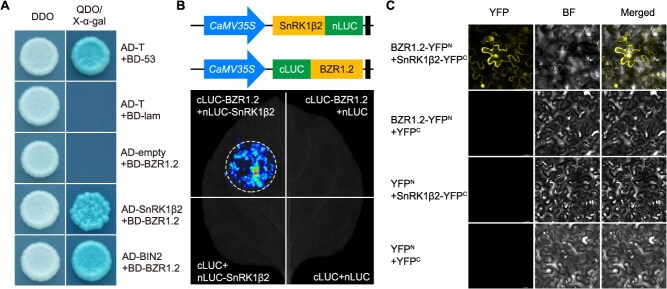
CaSnRK1β2 interacts with CaBZR1.2 at the protein level. (A) Y2H assay indicates the interaction between CaSnRK1β2 and CaBZR1.2. AD-BIN2 and BD-BZR1.2, AD-T and BD-53 serve as positive controls, AD-T and BD-lam serve as negative controls, respectively. (B) LCI assay confirms the interaction between CaSnRK1β2 and CaBZR1.2. (C) BiFC assay demonstrates the interaction between CaSnRK1β2 and CaBZR1.2 on the nucleus and cell membrane.

To further verify the interaction between CaSnRK1β2 and CaBZR1.2, we conducted targeted Y2H assays on a stringent selective medium (SD/-Trp-Leu-His-Ade) supplemented with X-α-galactoside. Meanwhile, CaBZR1.2 and CaBIN2 were cotransformed as a positive control. The results showed that CaSnRK1β2 interacted with CaBZR1.2 (Fig. 7A). In addition, we further verified the interaction between CaBZR1.2 and CaSnRK1β2 using LCI and BiFC assays (Fig. 7B and C). Collectively, these results demonstrated that CaBZR1.2 interacts with CaSnRK1β2.

### Silencing of *CaSnRK1β2* significantly promotes pepper lateral branch formation

**Figure 8:**
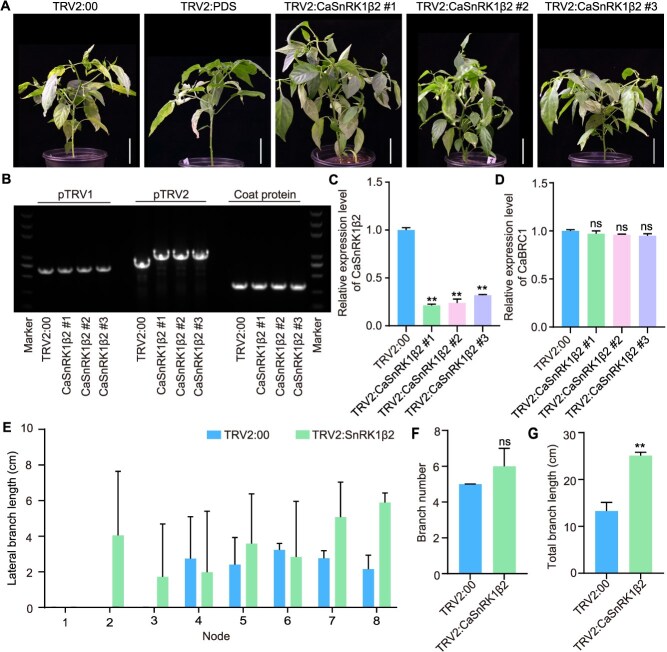
Silencing of the *CaSnRK1β2* gene promotes lateral branch growth in pepper. (A) The representative images of *CaSnRK1β2*-silenced plants, scale bars, 5 cm. (B) Identification of TRV based on PCR. The template was obtained by reverse transcription of total RNA from the node or lateral branch of negative control TRV2:00 and experimental groups TRV2:*CaSnRK1β2*. (C and D) The expression level of *CaSnRK1β2* and *CaBRC1* in control and *CaSnRK1β2*-silenced plants. (E-G) Average length of lateral branches at each node, number of lateral branches, and total length of lateral branches in control and *CaSnRK1β2*-silenced plants. Values are mean ± SD (*n* = 3).

To further verify the function of *CaSnRK1β2*, we silenced the *CaSnRK1β2* gene in ‘Zhangshugang’ pepper. The results showed that the lateral branches at each node of *CaSnRK1β2*-silenced lines became longer, and the total length of lateral branches significantly increased (Fig. 8A, B, E, and  G). The number of lateral branches slightly increased but without significant statistical difference (Fig. 8F). RT-qPCR was used to detect the expression levels of *CaSnRK1β2* and *CaBRC1* in stems and lateral branches; in silenced plants, the expression level of *CaSnRK1β2* decreased by 67.2%–80.3% compared with the control, while the expression level of *CaBRC1* showed no significant change (Fig. 8C and D). These results indicate that *CaSnRK1β2* can inhibit the initiation and elongation of lateral branches in pepper.

## Discussion

### Origin and evolution of the plant *BZR* gene family

The *BZR* family is a crucial transcription factor family in the BR signaling pathway and is widely distributed across plants [36–39]. However, the origin and evolutionary history of *BZR* remain unclear. In this study, we identified *BZR* family members from representative species ranging from algae to angiosperms. Previous studies have reported the occurrence of *BZR* in liverworts [40]. We found that the earliest algal species harboring *BZR* genes are charophytes, and multiple early clades had already emerged during the algal stage (Fig. 1). This finding is consistent with previous studies [41], which helps clarify the origin of this gene family. The number of *BZR* genes underwent significant expansion in pteridophytes, and the family size further increased in angiosperms. The copy number of *BZR* genes varied substantially among different species, ranging from five to 43. Most angiosperm species contained seven to nine *BZR* genes, with the highest number (43) detected in *T. turgidum*, while the lowest (5) was found in *F. nilgerrensis* and *B. vulgari*s.

With the evolution of plants from aquatic to terrestrial environments, the *BZR* family eventually formed five stable subgroups in angiosperms. The analysis of the evolutionary patterns of the *BZR* gene family across diverse plant lineages enhances our in-depth understanding of its phylogenetic relationships and evolutionary history. This result also highlights the necessity and advantages of conducting large-scale comparative analysis of gene families across the entire plant kingdom, providing important theoretical support and references for elucidating the origin and subsequent diversification of the *BZR* gene family.

### BZR1 plays an important role in regulating the development of lateral branches in pepper

The *BZR1* transcription factor plays a crucial role in plant growth, development, and stress response. Studies have shown that *BZR1* can regulate the growth of plant lateral branches. For example, OsBZR1 interacts with D53 to inhibit the expression of the tillering inhibitor *FC1*. This inhibition depends on OsBZR1 directly binding to DNA, which recruits D53 to bind to the *FC1* promoter in rice buds [28]. In addition, mutations in *BZR1* in tomatoes can inhibit bud growth, while overexpression of *BZR1* significantly promotes bud growth [42]. *BZR1* in rice and tomato shares similar functions and mechanisms with other species, but whether this is also true for pepper remains to be explored. In this study, we identified and characterized the function of *CaBZR1.2* (a homologous gene of *OsBZR1*). We found that silencing *CaBZR1.2* significantly reduced the number of lateral branches in peppers. This result indicates that *CaBZR1.2* promotes the occurrence and elongation of pepper lateral branches, which is consistent with previous studies. The overexpression of *CaBZR1.2* in tomato further confirmed our findings. Notably, in the silenced and OE plants, *CaBZR1.2* and *CaBRC1* exhibited opposite expression patterns (Figs 5C, D and 6D, E), suggesting that *CaBZR1.2* may function by inhibiting *CaBRC1*. Whether *CaBZR1.2* affects the branching process in peppers through *CaBRC1* warrants further investigation.

### CaBZR1.2 and the energy sensor CaSnRK1β2 antagonize the occurrence of lateral branches in pepper

SnRK1 is an evolutionarily conserved protein kinase complex, composed of catalytic α subunit (KIN10 and KIN11), regulatory β subunits (KINβ1, KINβ2, and KINβ3), and a plant-specific hybrid γ subunit (KINβγ), forming a heterotrimeric complex that regulates the energy homeostasis in plants [34, 43]. In *Arabidopsis*, the number of branches of *snrk1α1* mutants significantly increases, indicating that *SnRK1α1* negatively regulates branching [35, 44]. In rice, overexpression of *SnRK1α1* leads to an increase in tiller number and delayed flowering [45]. The transcription factor *bZIP11* inhibits branch formation, while Tre6P inhibits the accumulation of bZIP11 protein, thereby promoting branch formation. The SnRK1 also inhibits branch formation by alleviating the inhibitory effect of Tre6P on bZIP11 protein accumulation [44]. These results indicate that members of the SnRK1 family have a role in regulating branching/tillering. In this study, we found that CaSnRK1β2 can interact with CaBZR1.2 at the protein level. By silencing the *CaSnRK1β2* gene in peppers, the occurrence of lateral branches was distinctly different from that of *CaBZR1.2*. We also detected the expression of *CaBZR1.2* and *CaSnRK1β2* in silenced plants, and the results showed that gene silencing did not affect the transcription of interacting genes (Fig. S3A). Tissue-specific expression showed that *CaSnRK1β2* had the highest expression level in flowers (Fig. S2B), suggesting that *CaSnRK1β2* may influence lateral branch growth by controlling the transition between vegetative and reproductive growth. This process may involve the energy allocation of plants. A recent review article discusses sugar-regulated shoot branching and systematically describes the process of SnRK1-mediated branching [46]. Sugar may participate in the SnRK1-mediated branch growth process. This provides a clue for clarifying the relationship between *CaBZR1.2* and *CaSnRK1β2*. Our preliminary data suggest that CaSnRK1β2 and CaBZR1.2 antagonistic interaction regulates the lateral branch development and elongation of pepper. However, whether they function by stabilizing energy balance remains unknown.

### BIN2 may regulate branching by phosphorylating BZR1 and SnRK1β2

**Figure 9:**
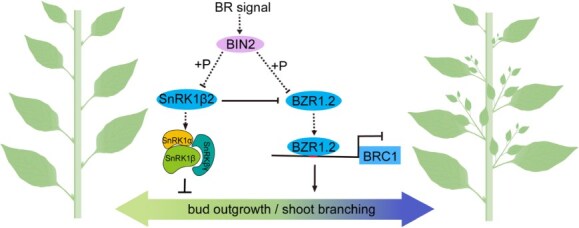
A proposed working model for BZR1.2 regulation of the lateral branch. The phosphorylation of SnRK1β2 by BIN2 weakens the activity of the SnRK1 complex, thereby reducing its ability to inhibit branching. BZR1.2 can also be phosphorylated by BIN2, which decreases its transcriptional repression activity on *BRC1*, thus inhibiting lateral branch formation. Additionally, SnRK1β2 may also inhibit the function of BZR1.2 through phosphorylation.

The GSK3-like kinase BIN2 is a crucial component of the BR signaling pathway, responsible for transmitting receptor signals to the nucleus. In the absence of BRs, BIN2 phosphorylates the BZR1 and BES1 proteins, resulting in the loss of their DNA-binding activity and eventual degradation by the 14-3-3 proteins in the nucleus. The PP2A phosphatase can dephosphorylate BES1/BZR1, allowing the nonphosphorylated BES1/BZR1 to initiate downstream gene expression, thereby regulating plant growth and development [34]. Studies have shown that *BIN2.2* is a significant inhibitor of bud growth in tomatoes; the author also found that the levels of transcripts encoding *BRC1* were decreased in the buds of the *bin2.2* [42]. BIN2 can phosphorylate SnRK1β2, thereby reducing its membrane-binding ability and its interaction with SnRK1α, further affecting the activity of SnRK1 [47]. Our RT-qPCR results show that after *CaBZR1.2* or *CaSnRK1β2* silencing, some genes of BR signaling pathway such as *CaBIN2* are affected to some extent (Fig. S3B). We speculate that the two may influence the downstream target genes to play their functions. Therefore, we speculate that the phosphorylation or dephosphorylation state of SnRK1β2 and BZR1.2 may be an important factor affecting lateral branch growth. These hypotheses provide new and important insights into the regulatory network of plant branching, which require further validation (Fig. 9).

## Conclusion

Our study investigated the origin and evolutionary trajectory of the *BZR* gene in plants, and identified the pepper *BZR1* pan-gene family, focusing on the mechanism of action of *CaBZR1.2*, and determined that *CaSnRK1β2* antagonizes *CaBZR1.2* to regulate lateral branch occurrence. In the future, we can control the number of branches by regulating the expression of *CaBZR1.2* and *CaSnRK1β2* during the breeding process of crops that require branching. To obtain more or fewer lateral branches, the expression of *CaBZR1.2* and *CaSnRK1β2* can be increased or decreased through overexpression or gene editing techniques.

## Materials and methods

### Identification of *BZR* family members in different species

The dataset of 78 species were retrieved from the public database PlantGIR (http://plantgir.cn/) and other public repositories, including Phytozome (https://phytozome-next.jgi.doe.gov/), CNGB (https://db.cngb.org/search/), NCBI (https://www.ncbi.nlm.nih.gov), Ensembl Plants (https://plants.ensembl.org/), TVIR (http://tvir.bio2db.com), and TBGR (http://www.tbgr.org.cn) [48–50], among others. These latest genomic data have been comprehensively collected and collated. Alternative splicing variants were removed using TBtools and Perl scripts to ensure the nonredundancy of the sequences utilized.

Candidate protein sequences were screened using the PF05687 domain from the Pfam (http://pfam.xfam.org/) and HMMER (v3.0) with an E-value cut-off of 1e^−5^, followed by refinement via BLAST+ alignment against *Arabidopsis* BZR reference sequences. Sequences containing the BES1_N domain were further filtered using the SMART database (https://smart.embl.de). To facilitate data processing and analysis, we added the corresponding species abbreviation prefix to the gene ID of each original genome. Python scripts were used to calculate the number of members in the *BZR* gene family. The sequence information is listed in Table S3.

### Phylogenetic analysis and evolutionary trajectory exploration

The protein sequences of 78 species were aligned using MAFFT (v7.526). The multisequence alignment results were further filtered with TrimAl (v1.5) to eliminate regions with low alignment quality and high gap ratios, yielding high-quality alignment results. A phylogenetic tree was constructed using FastTree with default parameters. Based on its topology, the tree was divided into distinct clades. By analyzing the statistical data of these clades, we mapped the evolutionary trajectory of the *BZR* gene family.

### The pan-genome analysis of the *BZR1* family in pepper

The HMM profile of the domain PF05687 (BES1_N domain) was used to scan 14 pepper pan-genomes via HMMER. The 14 pepper genomes included five cultivated species (*Capsicum annuum*, *Capsicum baccatum*, *Capsicum chinense*, *Capsicum frutescens*, *Capsicum pubescens*) and several wild species (*Capsicum chacoense*, *Capsicum rhomboideum*, *Capsicum eximium*, etc.). Genome data files were downloaded from PepperBase (http://www.bioinformaticslab.cn/PepperBase/), Pepper Genomics Database (http://ted.bti.cornell.edu/cgi-bin/pepper/index), PGDB (http://www.pepperbase.site/), and SolPGD (http://www.bioinformaticslab.cn/files/genomes/pepper_pan). Meanwhile, the six *Arabidopsis BZR1* members were used as seed sequences for BLASTp search that was conducted with an E-value cut-off of 1e^−10^. The results of HMMER and BLASTp were integrated, and sequences that were identified by both methods were retained. Subsequently, Pfam and NCBI CDD (https://www.ncbi.nlm.nih.gov) were used for dual verification to confirm that the candidate sequences contain complete *BZR1* family-conserved domains. Finally, nonredundant sequences were selected as potential pepper *BZR1* homologs. The sequence information is listed in Table S4.

A phylogenetic tree was constructed for the identified CaBZR1 protein sequences using the NJ method in MEGA 11 software, with 1000 bootstrap replicates. Visualization was implemented using the online tool tvBOT (https://www.chiplot.online/tvbot.html) [51]. The Ka/Ks values were calculated using KaKs_Calculator and visualized with TBtools software [52]. MCScanX software was used for collinearity analysis of BZR1 genes in pepper, *Arabidopsis*, rice, and tomato, with results visualized using TBtools software. MUSCLE was used for multiple sequence alignment, and visualization was performed with Jalview (http://www.jalview.org/). Motif analysis was conducted using the MEME online tool (https://meme-suite.org/meme/tools/meme), with the maximum number of conserved domains set to 10, minimum motif width set to 6, and maximum motif width set to 50.

### RNA extraction and RT-qPCR analysis

Total RNA was isolated using a plant RNA extraction kit (Cwbio, Taizhou, China). cDNA was synthesized from 1 μg of total RNA using a reverse transcription kit (Vazyme, Nanjing, China). RT-qPCR analysis was performed using SYBR Green (Vazyme, Nanjing, China) on a Roche LightCycler 96 real-time PCR system. The expression level of the target gene in pepper was normalized using *CaActin* [53] as the reference gene. The primers used are listed in Table S5.

### Subcellular localization

The full-length coding sequence of *CaBZR1.2*, excluding the stop codon, was cloned into the pCAMBIA1300-GFP vector to generate the recombinant plasmid pSuper:CaBZR1.2. Subcellular localization assays were performed using fully expanded young leaves from ~6-week-old *N. benthamiana* plants. Nuclear localization marker *AtH2B* [54], cloned into the pCAMBIA1300-mCherry vector, were coinfiltrated with the recombinant construct. The empty vector pCAMBIA1300-GFP was used as a positive control. Observations were conducted 2 days postinfiltration using a Leica confocal microscope (Leica DMI8, Wetzlar, Germany), with excitation/emission wavelengths set at 488/510 nm for GFP and 561/610 nm for mCherry. CaSnRK1β2 was analyzed using the same method. The primers used are listed in Table S5.

### Overexpression of *CaBZR1.2* in tomato plants

Using pepper cDNA as a template, the full-length sequence of the *CaBZR1.2* gene was amplified with gene-specific primers and cloned into the pU1301-Flag vector. Heterologous transformation of *CaBZR1.2* was performed in tomato cultivar Micro Tom. PCR analysis was used to verify the transformation status in transgenic plants, and RT-qPCR was employed to detect the expression level of *BZR1.2* in transgenic plants. A total of three homozygous T3 CaBZR1.2-OE tomato lines, namely OE#1, OE#2, and OE#3, were screened out for subsequent analysis. The primers used are listed in Table S5.

### Virus-induced gene silencing assay

Effective silencing targets of *CaBZR1.2* were analyzed via the Sol Genomics Network (https://solgenomics.net/), using the inbred line ‘Zunla-1’ as the template. This fragment was cloned into the *Sma*I restriction site of the pTRV2-C2b vector to generate the silencing vector pTRV2-*CaBZR1.2*. *Agrobacterium tumefaciens* (strain GV3101) harboring the pTRV2-*CaBZR1.2* and pTRV1 vectors, respectively, were coinfiltrated into 2-week-old seedlings of the inbred line ‘Zhangshugang’, which is a pepper line with abundant lateral branches. Plants coinfiltrated with pTRV2 and pTRV1 served as the negative control, while plants coinfiltrated with pTRV2-*PDS* and pTRV1 were used as the positive control. The plant growth conditions were set as follows: temperature 20°C–22°C, relative humidity 60%–70%, and photoperiod 16/8 h (light/dark). After inoculation, the number and length of lateral branches were recorded, and the stem nodes where lateral branches were located were collected for RT-qPCR analysis. The gene silencing procedure for *CaSnRK1β2* was the same as described above. The primers used are listed in Table S5.

### Measurement of lateral branch length

Digital Vernier calipers were used to measure the length of branches. Branches with a length ≥0.2 cm were considered as effective lateral branches, while buds with a length <0.2 cm were defined as lateral buds and excluded from the statistical scope of lateral branch number.

### Y2H assay

To evaluate protein–protein interactions, the full-length coding sequences of *CaBZR1.2* and *CaSnRK1β2* were cloned into the *Eco*RI/*Bam*HI sites of pGBKT7 and pGADT7 vectors, respectively, to construct pGBKT7-CaBZR1.2 and pGADT7-CaSnRK1β2. These constructs were then cotransformed into Y2H-Gold yeast competent cells, with pGBKT7-CaBZR1.2 paired with either pGADT7 or pGADT7-CaSnRK1β2. For control experiments, the pGADT7-T plasmid was cotransformed with either pGBKT7-53 or pGBKT7-lam, serving as positive and negative controls, respectively, using the same transformation method. Yeast transformants were initially selected on SD/-Leu/-Trp medium, and then transferred to SD/-Ade/-His/-Leu/-Trp medium for interaction testing with X-α-Gal. The primers used are listed in Table S5.

### Firefly LCI assay

The full-length coding sequences of *CaBZR1.2*, excluding the stop codon, and *CaSnRK1β2* were inserted into the pCAMBIA1300-cLUC and pCAMBIA1300-nLUC vectors, respectively. These constructs were transformed into *Agrobacterium*, mixed in a 1:1 ratio, and infiltrated into *N. benthamiana* leaves. At 2 days post-infiltration, 1 mM potassium luciferin was applied to the abaxial leaf surface, followed by a 5-min dark treatment for signal detection. Signal acquisition was performed using a plant *in vivo* imaging system (DynaPlant® Desktop, Beijing, China). The primers used are listed in Table S5.

### BiFC assay

The full-length coding sequences of *CaBZR1.2* and *CaSnRK1β2*, excluding the stop codon, were inserted into the pSPYNE-35S and pSPYCE-35S vectors, respectively. These constructs were transformed into *Agrobacterium*, mixed in a 1:1 ratio, and infiltrated into *N. benthamiana* leaves, with a small amount of p19 helper plasmid added. Approximately 3 days later, observations and imaging were performed using a Leica confocal microscope (Leica DMI8, Wetzlar, Germany). For the experiment, the excitation/emission wavelengths were set to 514 nm/527 nm for YFP. The primers used are listed in Table S5.

### Statistical analysis and data visualization

Significant differences between two groups were determined using Student’s *t*-test (^*^P < 0.05 and ^**^P < 0.01). When comparing three or more groups, one-way analysis of variance (ANOVA) was used, lowercase letters denote significant differences among groups at the 0.05 level. Data are reported as the mean ± SD. The 2^−△△Ct^ method was used for calculation of relative expression of genes. All statistical data and visualization analyses were carried out using GraphPad Prism 9.5.0 (Graph Pad Software, San Diego, CA, USA).

## Supplementary Material

Web_Material_uhag015

## Data Availability

The data that supports the findings of this study is available in the supplementary material of this article.
