# Supplementary figures and images for: Origin and evolution of BZR gene family in plants, pan-genome analysis of the BZR1 gene family, and functional characterization of CaBZR1.2 in pepper lateral branch development
Source: Hortic Res. 2026 Jan 20;13(4):uhag015. doi: 10.1093/hr/uhag015 (PMC13095355; doi:10.1093/hr/uhag015)

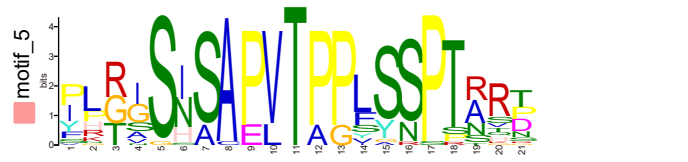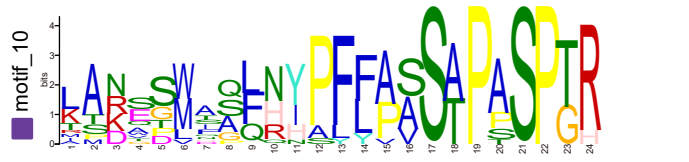

Supplement: Web_Material_uhag015 [file web_material_uhag015.zip › Fig. S1.pdf]

3

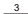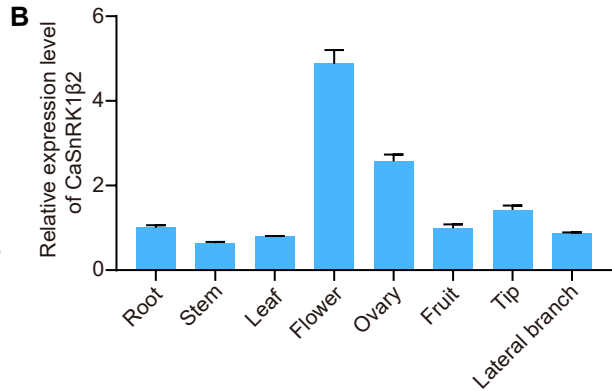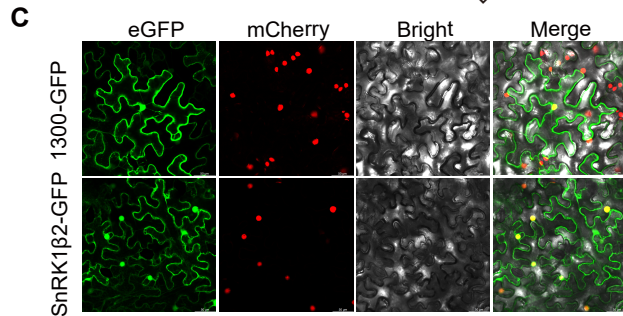

Supplement: Web_Material_uhag015 [file web_material_uhag015.zip › Fig. S2.pdf]

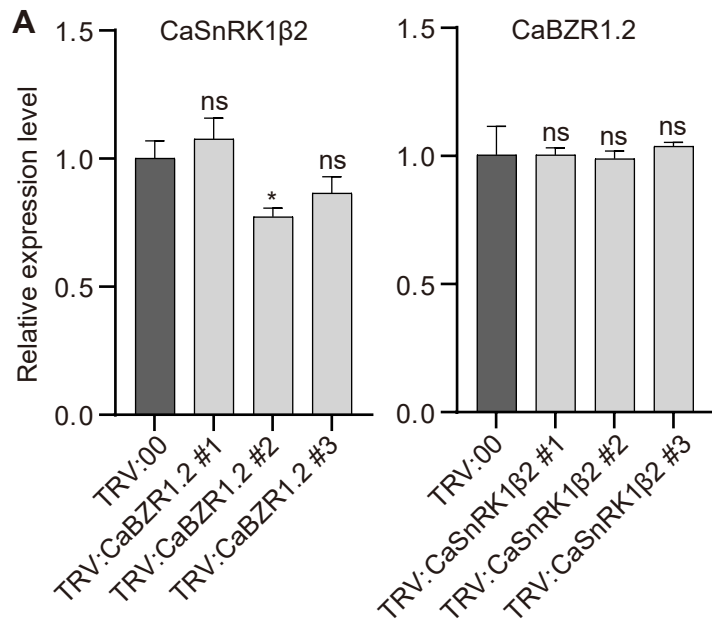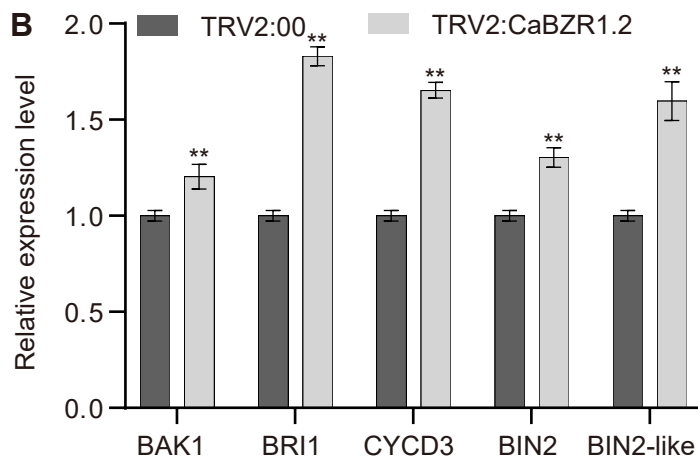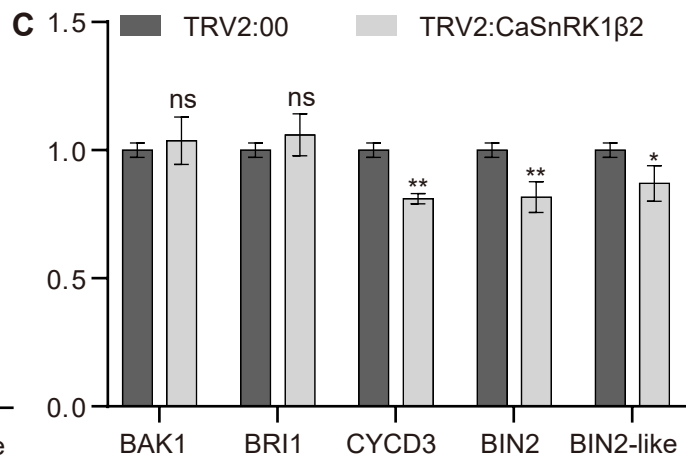

Supplement: Web_Material_uhag015 [file web_material_uhag015.zip › Fig. S3.pdf]
